# Self-Management Systems for Patients and Clinicians in Parkinson Care: Protocol for an Integrated Scoping Review, Product Search, and Evaluation

**DOI:** 10.2196/58845

**Published:** 2024-09-24

**Authors:** Selina Boege, Madison Milne-Ives, Edward Meinert, Camille Carroll

**Affiliations:** 1 Translational and Clinical Research Institute Faculty of Medical Sciences Newcastle University Newcastle upon Tyne United Kingdom; 2 Centre for Health Technology University of Plymouth Plymouth United Kingdom; 3 Department of Primary Care and Public Health School of Public Health Imperial College London United Kingdom; 4 University Hospitals Plymouth NHS Trust Plymouth United Kingdom

**Keywords:** Parkinson’s disease, digital health, self-management, health care systems, self-care, Parkinson, mobile health, mHeath, evaluation, acceptability, usability, decision-making support, database, qualitative, quantitative, mixed method, perception

## Abstract

**Background:**

Parkinson disease (PD) poses emotional and financial challenges to patients, families, caregivers, and health care systems. Self-management systems show promise in empowering people with PD and enabling more control over their treatment. The collaborative nature of PD care requires communication between patients and health care professionals. While past reviews explored self-management systems in PD diagnosis and symptom management with a focus on patient portals, there is limited research addressing the interconnectivity of systems catering to the needs of both patients and clinicians. A system’s acceptability and usability for clinicians are pivotal for enabling comprehensive data collection and supporting clinical decision-making, which can enhance patient care and treatment outcomes.

**Objective:**

This review study aims to assess PD self-management systems that include a clinician portal and to determine which features enhance acceptability and usability for clinicians. The primary aim is to assess evidence of clinicians’ acceptability and usability of self-management systems with a focus on the integration of systems into clinical workflows, data collection points, monitoring, clinical decision-making support, and extended education and training.

**Methods:**

The review will entail 3 separate stages: a literature review following the PRISMA-ScR (Preferred Reporting Items for Systematic Reviews and Meta-Analyses extension for Scoping Reviews) guidelines, a product search, and an evaluation of the level of evidence for the identified products. For the first stage, 5 databases will be searched: PubMed, CINAHL, Scopus, ACM digital library, and IEEE Xplore. Studies eligible for inclusion will be qualitative, quantitative, and mixed methods studies examining patients’ and clinician’s perceptions of the acceptability and usability of digital health interventions, synthesized by a narrative qualitative analysis. A web search in the iOS Apple App Store and Android Google Play Store will identify currently available tools; the level of evidence for these will then be assessed using the Oxford Centre for Evidence-Based Medicine guidelines.

**Results:**

Literature search and screening began soon after submission of the protocol, and the review is expected to be completed by end of September 2024.

**Conclusions:**

This review will examine currently available self-management systems in PD care, focusing on their acceptability and usability. This is significant because there is limited research addressing the integration of clinicians into these systems. The findings from this study may provide critical knowledge and insight to help inform future research and will contribute to the design of self-management systems that promote collaborative efforts in PD care.

**International Registered Report Identifier (IRRID):**

PRR1-10.2196/58845

## Introduction

Parkinson disease (PD) is a complex and progressive neurological condition with no current cure. It represents the second most common neurodegenerative disorder worldwide [1], with a 30% rise in prevalence and incidence between 2018 and 2030 [2,3]. Clinicians in PD care often have limited data due to infrequent patient contact, constraining their ability to fully inform treatment decisions [4]. The diversity of symptoms experienced by people with PD means that care management is complex. Optimizing symptom management requires an understanding of current symptoms and trends, necessitating thorough communication between patients and clinicians [4,5]. Positive relationships between patients and clinicians can enhance patient trust, medication adherence, and the patients’ active engagement in care, with potential benefits in mitigating health care disparities, fostering interdisciplinary collaboration, and ultimately enhancing patient outcomes [6,7]. Given the strain on health care resources and the limited regular clinical contacts, digital technologies have the potential to help patients self-manage symptoms and provide accurate records of symptoms for clinical review. Despite the availability of such systems, there is limited understanding of how they are adopted, accepted, and used within clinical contexts.

Despite advancements in digital health, many systems still fall short in effectively compiling information for clinician use at the point of care and facilitating seamless care communication [6]. Systems that combine patient self-reporting with clinician management can improve data collection and communication, leading to more efficient care, better decision-making, and ultimately enhanced patient outcomes through more timely and personalized interventions [6], [7]. Previous research has extensively examined engagement with and impact of digital tools in PD care, focusing on self-management methods to enhance physical and cognitive aspects for patients [8-18]. A comprehensive understanding of the acceptance and usability by health care professionals (HCPs) is lacking, leaving room for more inclusive research in PD care [19-28]. There is a need to understand what systems are currently available, how they integrate self-management techniques, and how clinical elements and clinicians are integrated [29-35]. This knowledge gap is particularly crucial to address, as improving clinician engagement with digital tools can lead to more effective and tailored patient care strategies.

This study aims to fill this gap by examining the state of the literature on clinician perspectives of PD self-management systems and the state of clinician-focused features in existing self-management systems. The review will summarize available systems and evidence of their acceptability and usability of self-management systems, while the product evaluation will identify active digital applications, tools, and services (eg, apps, websites, portals, and wearable devices) that support self-management for people with PD to explore what types of technologies are used, which self-management techniques are applied, and how clinicians are integrated.

## Methods

### Design

This review is structured in 3 distinct stages, each contributing to building a comprehensive overview and understanding of the landscape of self-management systems for PD. We will perform a scoping review by leveraging bibliographic databases, search and select eligible systems on the Apple App store and Google Play store, and evaluate the level of their evidence. Identified self-management systems will be evaluated using the Oxford Centre for Evidence-Based Medicine (OCEBM) guidelines following adherence to established eligibility criteria. Separate analyses will be conducted for the scoping review and product evaluation, as they will extract different types of data, but findings from each will be synthesized in the discussion. The design concludes with a narrative synthesis to summarize study findings and product evaluation results, incorporating study methods, key findings, and outcomes. All review stages are summarized in Table 1.

**Table 1:** Review stages.

Review stage	Data collection method	Analysis method
Stage 1: a scoping review	Search bibliographic databases (PubMed, CINAHL, Scopus, ACM Digital Library, and IEEE Xplore) using PRISMA-ScR^a^ and PICOS^b^ frameworks	Narrative synthesis to identify key themes and gaps
Stage 2: product search	Search Apple App Store and Google Play Store using relevant keywords	Evaluation of system characteristics and user feedback
Stage 3: data analysis	Extract data from the literature review and product search	Adapt OCEBM^c^ guidelines for system classification and narrative synthesis

^a^PRISMA-ScR: Preferred Reporting Items for Systematic Reviews and Meta-Analyses extension for Scoping Reviews.

^b^PICOS: Population, Intervention, Comparator, Outcome, and Studies.

^c^OCEBM: Oxford Centre for Evidence-Based Medicine.

### Search Strategy

#### Stage 1: Scoping Review

##### Search

The first stage of the research involves the exploration of existing literature through 5 bibliographic databases: PubMed, CINAHL, Scopus, ACM Digital Library, and IEEE Xplore. The search strategy is designed to identify studies using qualitative, quantitative, and mixed method approaches that investigate HCPs’ perspectives on the acceptability and usability of self-management systems. The PRISMA-ScR (Preferred Reporting Items for Systematic Reviews and Meta-Analyses extension for Scoping Reviews; Multimedia Appendix 1) and Population, Intervention, Comparator, Outcome, and Studies (PICOS) frameworks were used to structure the protocol for the review strategy (Table 2) [35]. The PICOS framework was used to outline key themes that shaped the search strategy (Table 3), with themes relating to the population (HCPs), intervention (PD and self-management interventions), and outcomes (evaluation).

The scoping review will be used to systematically chart and synthesize data to identify key themes and gaps in the existing literature, thereby providing a comprehensive overview of this research results and studies to build the foundation for subsequent stages of this research, ensuring that relevant aspects are actual and addressed. The database search will be performed in August 2024 using PubMed (MeSH [Medical Subject Headings] terms), CINAHL (abstract), Scopus (title and abstract), ACM digital library (full text), and IEEE Xplore (full text). A preliminary exploration of identified relevant terms based on the PICOS framework was used to develop the search strategy (Multimedia Appendix 2). The search strategy will include a set of keywords relating to digital self-management tools and their user-friendliness and user acceptance. Keywords were derived from MeSH related to the subject and used as search terms (Table 3).

**Table 2:** PICOS^a^ framework [35].

PICOS	Detail
Population	Health care professionals treating patients
Intervention	Digital interventions for self-management for people with PD^b^, for example, telehealth, exergaming, websites, smart homes, mobile apps, web-based systems, and wearable devices, which offer a clinician portal or similar which may enable clinical use (eg, e-record integration, messaging features, remote monitoring, or treatment decision support)
Comparator	None
Outcomes	Primary outcome: clinician usability and acceptability of digital interventions for self-management in PD care ‎Secondary outcomes: factors that might impact the usability of these systems including regulatory requirements, training protocols, and any other elements that may influence the overall user experience for both people with PD and clinicians
Study types	Qualitative and quantitative studies, for example, case-control studies, case series, longitudinal studies, cohort studies, or RCTs^c^

^a^PICOS: Population, Intervention, Comparator, Outcome, and Studies.

^b^PD: Parkinson disease.

^c^RCTs: randomized controlled trials.

**Table 3:** MeSH^a^ terms and keywords used for literature search.

Category	MeSH terms	Keywords (in title and abstract)
Parkinson disease	Parkinson disease OR parkinsonian disorders	“Parkinson’s disease” OR parkinson OR parkinson’s OR “parkinson disease”
Health care professional	Physicians OR health personnel	“Healthcare professional” OR “healthcare personnel” OR clinician OR practitioner OR doctor OR nurse OR “care professional”
Self-management intervention	Electronic OR technology OR data collection OR internet-based intervention OR digital health OR telemedicine OR computing methodologies OR software OR wearable electronic devices OR self-help devices OR rehabilitation OR computer-user training	“Digital intervention” OR technology OR system OR portal OR remote OR home-based OR database management system OR internet-mediated therapy OR remote consultation OR personal health services
Evaluation	Quality of health care OR health care evaluation mechanism OR program evaluation OR attitude OR behaviour OR acceptance process OR acceptance processes OR treatment adherence and compliance OR communication methods, total OR security measures OR educational measurement OR time management OR efficiency, organizational	Evaluation OR attitude OR user experience OR acceptability OR usability OR impact OR acceptance OR compliance OR conformity OR efficiency

^a^MeSH: Medical Subject Headings.

##### Eligibility

Studies eligible for inclusion are papers that analyze self-management systems for people with PD and their HCP, embed clinical measures, or focus on clinical evidence of acceptability and usability. A comprehensive definition of self-management interventions was used to enable the collection of various intervention types. Any digital form of intervention, whether websites or mobile apps, are considered for inclusion if the system’s intention includes enhancing any aspect of patient self-management in PD. Inclusion criteria for literature encompass published studies at any given time, including randomized controlled trials, cohort studies, and case-control studies. There will be no limit of the publication date as this review aims to have a full overview of available self-management systems for patients with PD.

Studies that do not review or analyze self-management intervention for PD will be excluded as well as editorials, perspective articles, conference papers, and protocols. Literature that is not published in English will be excluded as the research team does not have the necessary resources to assess these.

##### Screening and Selection of Studies

The selection of studies will be determined by a review of study titles and abstracts in the indexed databases to assess their relevance to the use of digital self-management systems for PD. Selected references will be managed, and duplicates will be eliminated using the citation management software EndNote X21 (Clarivate). The initial screening of these references, based on search strategy keywords, will be conducted through EndNote X21’s search function. A total of 2 authors will screen the title and abstract of each record and will either include or exclude using the eligibility criteria previously described. Once the title and abstract screening is completed, selected records will be obtained for full-text screening. Full-text screening will be undertaken following the eligibility criteria. The stages of the scoping review process and selection of records will be presented using the PRISMA-ScR flowchart [35]. The data extraction and analysis process for this scoping review are described in stage 3.

#### Stage 2: Product Search

##### Search

The second stage includes the search and selection of digital interventions currently available in app stores. The process will be executed by searching the Apple App store and Google Play store using relevant keywords to identify and assess digital interventions. The keywords “Parkinson” and “Parkinson’s Disease” will be searched separately on both app stores; all results from each search will be extracted, the lists will be combined, and duplicates will be removed before screening. This stage is designed to encompass a diverse range of self-management tools available that are specific to PD or of potential relevance for patients managing specific symptoms caused by PD. This stage aims to respect the dynamic nature of the systems available for clinicians, ensuring the inclusion of real-world offerings to facilitate a holistic analysis of practical implications for available digital solutions.

##### Eligibility

Any digital self-management intervention, such as websites, web portals, mobile apps, or wearable devices, will be considered eligible if it primarily focuses on providing self-management services for individuals diagnosed with PD. The system will be designed to operate as a stand-alone product, eliminating the need for the combination of multiple devices. This was chosen to account for operational or financial consequences for the patient in acquiring the system. The system may adopt a paid subscription model, feature in-app purchases, or be offered free of charge to users. Systems included for review must be available in the Apple App store or Google Play store and accessible in English and in the United Kingdom. Eligible apps are any systems for the use of PD self-management. Detailed eligibility criteria are presented in Textbox 1.

**Eligibility criteria for systems on app stores included in the study.**

- Inclusion criteria
  - Systems available on the UK Apple App store or UK Google Play store
  - Systems available in English
  - Systems intended for the self-management of Parkinson disease
- Exclusion criteria
  - Systems not available on the UK Apple App store or UK Google Play store
  - Systems not available in English
  - Systems not yet released for public use or access

##### Screening and Selection of Self-Management Systems

All self-management systems found in the scoping review or product searches will be documented in a Microsoft Excel file, and duplicate entries will be removed. Systems meeting the criteria will be accessed and purchased, if necessary, and downloaded to either the MacOS system or iOS or Android devices. Any systems that, upon closer inspection, do not meet the inclusion criteria will be excluded. In case of disagreements between the reviewers, these will be discussed and, if required, resolved by a third reviewer.

#### Stage 3: Data Analysis

##### Data Extraction

Following completion of the screening process and deduplication using EndNote 21 software, eligible studies will be transferred to a Microsoft Excel spreadsheet for further data extraction (Textbox 2). Any modifications to the data extraction items will be recorded contemporaneously and reported in the final review.

**Full data charting list.**

-
  Literature: system description
  Name
  Year of launch
  Technology domain (eg, internet of things [IoT], mobile app, or website)
  Costs
  Evaluation method
  Type of self-management intervention
  Description of self-management intervention
- Product evaluation: system characteristics
  Intervention outcome or intention
  Possibility of data exchange (clinician-patient)
  Peer collaboration (clinician-clinician)
  Feedback mechanism
  Possibility of uploading eHealth records
  (Real-time) remote monitoring
  Alerts and notifications
  Training support
  Appointment scheduling
  Integration of wearables

##### Data Analysis

Data extracted from the studies included in the literature review will be analyzed and synthesized narratively. The criteria used to assess the systems found through literature and product searches will be adapted from the OCEBM guidelines [7]. In this categorization, systems will be classified based on the strength of their evidence, ranging from case reports to randomized controlled trial methodology. We will characterize self-management systems described in these reports based on different types of technology, such as mobile apps, websites, web portals, telehealth, digital communication platforms, digital solutions for data management, wearable devices, and web-based training systems. The analytical narrative synthesis of the reviewed literature will examine what self-management techniques are used within the systems and how clinicians are incorporated, investigating if their perspectives are solicited and the degree to which such feedback influences the design and evolution of these digital tools. Systems will be summarized focusing on clinical and operational intention to identify key trends and outcome measures will be examined. The results of this will be tabulated to facilitate visualization of identified systems and their characteristics and elaborated in the discussion of the review. Following the summarization of the findings from the literature review and system selection, the final stage will be a synthesis of qualitative findings from eligible studies combined with information derived from the system search and system descriptions or reviews on the app stores. By categorizing systems based on the technology domain and examining the qualitative data about user experiences, stage 3 aims to synthesize information found; this will enabling a deeper understanding of digital self-management solutions for patients and clinicians in relation to the type and features of the technology, to ensure that the systems not only serve patients but also include integral components in the clinical workflow, thus fostering an efficient and collaborative care environment. The synthesis of qualitative insights with system evaluations will reveal how well current tools support this vital relationship, guiding the development of future solutions to better meet the needs of both patients and HCPs.

### Ethical Considerations

There are no ethical considerations for the first stage (scoping review), as this will only examine previously published data. For the stage-2 production evaluation, we will be evaluating intervention features and will not have access to any patient data. The privacy of any user data accessed from app store reviews will be protected through anonymization in the data extraction process.

## Results

Literature search and screening began soon after submission of the protocol, and the review is expected to be completed by end of September 2024.

## Discussion

This review will examine currently available self-management systems within PD care with a particular focus on their acceptability and usability. The review will adopt a 3-stage approach, involving a scoping review, product search, and evidence of evaluation across various databases.

In the scoping review, there is also a potential publication bias, as the narrative synthesis may rely on published literature that predominantly reports positive outcomes, potentially overlooking negative or inconclusive results. In addition, selection bias could arise from the criteria used to assess and include systems, potentially favoring certain types of technology or studies with more rigorous methodologies. Finally, reporting bias might affect the qualitative synthesis, as the perspectives of clinicians and users reported in app store reviews or system descriptions may not fully capture the range of experiences, particularly those of individuals who did not engage with the tools or had negative experiences. To mitigate these biases, we will include a comprehensive range of studies, apply rigorous inclusion criteria, and seek diverse sources of qualitative data to ensure a balanced and representative analysis. Potential biases in data selection and interpretation may arise from the single-reviewer methodology, which is due to time and resource constraints.

Choosing only the Apple App store and Google Play store can be limiting, especially for health-related apps, as it may exclude specialized or region-specific app stores that offer unique or locally relevant health solutions. In addition, some innovative or niche health apps might only be available on alternative platforms like the Amazon App store, Samsung Galaxy Store, or F-Droid, potentially overlooking valuable resources for comprehensive health management.

The scope of the study will include an examination of the presence and extent of clinician-focused evaluations in existing research but will not deeply synthesize factors affecting clinicians’ acceptability and usability. This is a gap in the literature, but it is not within the scope of this review due to resource and logistical limitations, and the purpose of the scoping review is to provide an overview of the literature. Understanding how to best support clinician adoption and use will be an important factor in the implementation of such systems and a good target for further research.

Despite the limitations, the review is expected to contribute to the literature by addressing a gap in research on the acceptability and usability of self-management systems for clinicians. It will provide a comprehensive summary of self-management systems adopting different types of technology used in PD care and differentiate between patient-centered and clinician-centric tools, aiming to offer recommendations that can inform future system designs.

## Appendix

Multimedia Appendix 1 PRISMA-ScR (Preferred Reporting Items for Systematic review and Meta-Analysis Extension for Scoping Reviews) 2015 checklist.

Multimedia Appendix 2 Search strings of sample search.

## Abbreviations

HCP: health care professional
OCEBM: Oxford Centre for Evidence-Based Medicine
PD: Parkinson disease
PICOS: Population, Intervention, Comparator, Outcome, and Studies
PRISMA-ScR: Preferred Reporting Items for Systematic Reviews and Meta-Analyses extension for Scoping Reviews
