# Supplementary material for: Self-Management Systems for Patients and Clinicians in Parkinson Care: Protocol for an Integrated Scoping Review, Product Search, and Evaluation
Source: JMIR Res Protoc. 2024 Sep 24;13:e58845. doi: 10.2196/58845 (PMC11462133; doi:10.2196/58845)
Supplement: Multimedia Appendix 2 [file resprot_v13i1e58845_app2.docx]

**Multimedia Appendix 2**

Table S1: Search strings of sample search

| Database | Search String | Retrieved |
| --- | --- | --- |
| PubMed | (((("Parkinson disease"[MeSH Terms]) OR ("parkinsons") OR ("Parkinsonism" OR ("parkinson's") OR ("parkinsonian disorders"[MeSH Terms])) AND ((telemedicine[MeSH Terms]) OR ("internet-based intervention") OR ("digital health") OR ("remote") OR ("home-based") OR ("electronic") OR ("technology") OR ("software") OR ("m-health") OR (""computing methodologies") OR ("system") OR ("self-management system") OR ("portal") OR ("computing methodologies"[MeSH Terms]) OR ("e-health") OR ("wearable electronic devices"[MeSH Terms]) OR ("self-help devices"))) AND (evaluation) | 6,041 |
| CINAHL | AB ( parkinson disease or parkinson and disease or parkinson disease or parkinson’s disease ) AND TX ( software or system or remote or portal or technology or digital health or telemedicine or telehealth or ehealth or e-health or mhealth or m-health ) AND TX ( evaluation or analysis or perspective or attitude or user-experience or acceptability or usability or perspective or UX or barriers or perception ) | 5,172 |
| Scopus | TITLE-ABS-KEY ( ( "Parkinson disease" OR "parkinsonian disorders" OR "parkinsons" ) AND ( "telemedicine" OR "digital health" OR "internet-based intervention" OR "remote" OR "home-based" OR "wearable electronic devices" OR "computing methodologies" OR "electronic" OR "technology" OR "software" OR "m-health" OR "system" OR "portal" OR "e-health" OR "self-help devices" ) AND ( "attitude" OR "user-experience" OR "acceptability" OR "usability" OR "perspective" OR "UX" OR "barriers" OR "perception" ) ) | 7,820 |
| ACM digital library | [[Full Text: "parkinson disease"] OR [Full Text: "parkinson's disease"] OR [Full Text: "parkinsonian disorders"]OR [Full Text: "parkinsons"] OR [Full Text: "parkinson"]] AND [[Full Text: "telemedicine"] OR [Full Text: "digital health"] OR [Full Text: "internet-based intervention"] OR [Full Text: "remote"] OR [Full Text: "home-based"] OR [Full Text: "wearable electronic devices"] OR [Full Text: "computing methodologies"]OR [Full Text: "electronic"] OR [Full Text: "technology"] OR [Full Text: "software"] OR [Full Text: "m-health"] OR [Full Text: "system"] OR [Full Text: "portal"] OR [Full Text: "e-health"] OR [Full Text: "self-help devices"] OR [Full Text: "mhealth"]] AND [[Full Text: "evaluation"] OR [Full Text: "attitude"] OR [Full Text: "user-experience"] OR [Full Text: "acceptability"] OR [Full Text: "usability"] OR [Full Text: "perspective"] OR [Full Text: "ux"] OR [Full Text: "barriers"] OR [Full Text: "perception"]] | 1,855 |
| IEEE Xplore | ("All Metadata":"Parkinson disease" OR "All Metadata":"parkinsonian disorders" OR "All Metadata":"parkinsons") AND ("All Metadata":telemedicine OR "All Metadata":digital OR "All Metadata":remote OR "All Metadata":internet OR "All Metadata":electronic OR "All Metadata":technology OR "All Metadata":software OR "All Metadata":system OR "All Metadata":portal OR "All Metadata":"e-health" OR "All Metadata":"m-health" OR "All Metadata":"self-help devices" OR "All Metadata":"internet-based intervention" OR "All Metadata":"remote") AND ("All Metadata":evaluation OR "All Metadata":attitude OR "All Metadata":user OR "All Metadata":acceptability OR "All Metadata":usability OR "All Metadata":perspective) | 222 |
